# Supplementary material for: Transcriptome and Hormone Comparison of Three Cytoplasmic Male Sterile Systems in Brassica napus
Source: Int J Mol Sci. 2018 Dec 12;19(12):4022. doi: 10.3390/ijms19124022 (PMC6321506; doi:10.3390/ijms19124022)
Supplement: Supplementary file 1 [file ijms-19-04022-s001.pdf]

Table S1. Primer sequences for RT-PCR.

Gene-name	Primer-name	Primer sequence	Annotation
BnUnng1000260	1-F	ATGAGCTTCTCTTACAATCCTA	NAC transcription factor 56-like
	1-R	TGAGACGGTACTCATGCATGAT	
BnA09g0350100	2-F	TCGGTTTCATCCGACCGACGA	PREDICTED: NAC transcription factor 25
	2-R	TAGTGAGTTTTTCCTTGACGT	
BnA01g0035970	3-F	TCATCCAACCGATGAAGAGCT	PREDICTED: NAC transcription factor 56-like
	3-R	TTCTTGTTGCCGTAATCACAT	
BnC07g0816730	4-F	AGGTTCTTGACATCAGCTTCTC	PREDICTED: NAC domain-containing protein 100
	4-R	AATGTTCTTGACCAAGAATGGG	
BnA02g0071160	5-F	TTACGTAACGTTTCGTCGTATT	PREDICTED: bZIP transcription factor 44-like
	5-R	CAGAGTATCACATCATTCATAT	
BnC03g0540620	6-F	CAGATCAAAGATGTCTAGA	PREDICTED: protein TIFY 9-like isoform X1
	6-R	AGTCCCAATCAACAAAGAG	
BnC01g0467060	7-F	GCAACAAGCAAAAGGTGAA	PREDICTED: transcription factor MYB26-like isoform X1
	7-R	TTGTGTTGGTAGTGGTTAGA	
BnC01g0439330	8-F	ACGTCAAAATGGAGAGTTCA	PREDICTED: probable WRKY transcription factor 31
	8-R	TTCTGTTTCCGTTGTTGTTG	
BnA06g0242820	9-F	TGGTCAAAATCATCCTAACCCT	PREDICTED: LOB domain-containing protein 27
	9-R	TCTCAATCAATTCACACTCGTG	
BnA01g0035930	10-F	GTGTACCTTACTTGAGGAAGAT	PREDICTED: auxin-responsive protein IAA19-like isoform X1
	10-R	GTCTTCGTATATGGTAACGTAT	
BnUnng1000190	11-F	GCCGCCGGTTTGCTCTTACCGG	PREDICTED: auxin-responsive protein IAA19-like isoform X1
	11-R	GTCTTCGTATATGGTAACGTAT	
BnA08g0306020	12-F	GTAGCCGGATTCCGTCGTAGCT	PREDICTED: abscisic acid 8'-hydroxylase 3
	12-R	TCTCCGAGTATTGAGATTAACG	
BnC09g0904760	13-F	TAGGCGAAACGTTCCAGCTTTA	PREDICTED: abscisic acid 8'-hydroxylase 3-like
	13-R	GATACTCTGTTTTTAGACTGAG	
Actin	A-F	TCTGGCATCACACTTTCTACAACGAGC	Internal reference for quantification of gene expression
	A-R	CAGGGAACATGGTCGAACCACC	
